# Feasibility, Acceptability, and Initial Outcomes of a Yoga-Based Group Intervention for Inpatients with Schizophrenia Spectrum Disorders: A Rater-Blinded Randomized Controlled Trial

**DOI:** 10.1192/j.eurpsy.2025.2219

**Published:** 2025-08-26

**Authors:** I. Hahne, M. Zierhut, N. Bergmann, E. Hahn, T. M. T. Ta, C. Calvano, M. Bajbouj, K. Böge

**Affiliations:** 1 Department of Psychiatry and Psychotherapy, Charité – Universitätsmedizin Berlin; 2 German Center of Mental Health (DZPG); 3 BIH Charité Clinician Scientist Program, Berlin Institute of Health; 4 Department of Education and Psychology, Clinical Child and Adolescent Psychology and Psychotherapy, Freie Universität Berlin, Berlin; 5 Medical University Brandenburg, Brandenburg, Germany

## Abstract

**Introduction:**

The efficacy of yoga as an adjunctive treatment for schizophrenia spectrum disorders (SSD) has garnered interest, however, meta-analytic findings exhibit heterogeneity. While yoga may positively influence various symptom domains, further investigation is needed due to the limited number, quality, and generalizability of studies. Yoga-based Group Intervention (YoGI) was specifically developed together with persons with SSD through a participatory approach and its mechanisms and processes were explored within qualitative studies.

**Objectives:**

This pre-registered randomized controlled trial (RCT) assessed the acceptability and feasibility as well as preliminary outcomes of YoGI compared to a commprehensive treatment as usual (TAU) in an inpatient setting.

**Methods:**

Fifty inpatients with SSD received either treatment as usual (TAU, *n* = 25) or YoGI+TAU (*n* = 25) for four weeks. Preliminary analyses examined rater-blinded positive and negative symptoms, self-rated depressive and anxiety symptoms, body mindfulness, mindfulness, psychological flexibility, subjective cognition, social functioning, quality of life, and medication regime at baseline and post-intervention.

**Results:**

Outcomes showed a 95% protocol adherence, feasibility and retention rates of 91% and 94%, respectively, and a dropout rate of 6%. ANCOVA revealed significant between-group post-intervention improvements for YoGI+TAU in positive symptoms, depression, cognitive fusion, and a mindfulness subscale. Medium-to-large pre-to-post intervention effects were found for body-mindfulness, positive, negative, and general symptomatology, depression, anxiety, stress, cognitive fusion, attention, and quality of life in YoGI+TAU, while within-group changes were consistently smaller in TAU. No severe adverse events were reported.

**Conclusions:**

This trial contributes to the growing evidence supporting the feasibility and acceptability of yoga for persons with SSD in an inpatient setting. Furthermore, preliminary evidence suggests that YoGI may provide additional benefits beyond TAU alone, across various self- and rater-based outcomes. These outcomes include improvements in body mindfulness, mindfulness, and psychiatric symptomatology, including positive and negative symptoms, subjective cognition, depression, anxiety, stress, social functioning, and quality of life. Additional fully powered RCTs are warranted to further elucidate the efficacy and potential mechanisms of action of YoGI for SSD, which should also assess the cost-efficiency of YoGI and explore longitudinal changes associated with the intervention. Such comprehensive research endeavours will not only enhance our understanding of the therapeutic potential of YoGI but also inform clinical practice and intervention strategies for persons with SSD.

**Disclosure of Interest:**

None Declared

